# Effect of short‐term dynamic psychotherapy on sexual function and marital satisfaction in women with depression: Clinical trial study

**DOI:** 10.1002/hsr2.1370

**Published:** 2023-06-23

**Authors:** Arash Ziapour, Arezou Hajiazizi, Maryam Ahmadi, Fateme Dehghan

**Affiliations:** ^1^ Cardiovascular Research Center, Health Institute, Imam‐Ali Hospital Kermanshah University of Medical Sciences Kermanshah Iran; ^2^ Department of Reproductive Health, Faculty of Nursing and Midwifer Kermanshah University of Medical Sciences Kermanshah Iran; ^3^ Faculty of Medicine Kermanshah University of Medical Sciences Kermanshah Iran

**Keywords:** depression, intensive short‐term psychotherapy, marital satisfaction, sexual function, women

## Abstract

**Background and Aims:**

This study aimed to look at the influence of short‐term dynamic psychotherapy on sexual function and marital satisfaction in women with depression.

**Methods:**

Through a clinical trial study using a pretest–posttest design and a control group, this study enlisted the participation of 60 women diagnosed with depression. The patients were interviewed before being randomly assigned to experimental or control groups. Data were obtained through the Beck Depression Inventory, the Enrique Marital Satisfaction Questionnaire, and the Female Sexual Function Questionnaire. The experimental group received intense short‐term dynamic psychotherapy intervention, while the control group was on a 2‐month waiting list. The SPSS 24 program utilized an analysis of variance to analyze the data.

**Results:**

The pre‐ and posttest study results revealed a significant difference in marital satisfaction, sexual function, and depression between the experimental and control groups (*p* < 0.01).

**Conclusion:**

During the posttest phase, a short‐term intensive dynamic psychotherapy intervention helped the experimental group feel better about their marriage and improve their sexual function. It also helped them feel less depressed.

## 1 INTRODUCTION

Marriage provides a range of psychological and physical requirements in a safe environment, and it has the potential to have a significant impact on people's mental health.^1^ There is a link between having efficient sexual relationships and having a happy marriage, and this link might be one of the most significant predictors of marital satisfaction.^2^, ^3^ If a sexual activity does not meet a person's requirements, it may lead to emotions of deprivation, failure, and loss of safety, which can jeopardize a person's mental health and eventually lead to the breakup of their family.^4^

As a psychological term, marital happiness may be measured on a scale ranging from low to high in many elements of a married relationship, including communication, personality differences, conflict resolution, sexual interactions, and children.^5^, ^6^, ^7^

People who engage in acrimonious relationships with one another are often overpowered by unpleasant sentiments. The vast majority of patients seek therapy because they are unable to channel their emotions into constructive forms of expression and activity. As a consequence, people get worried and resort to self‐defense. As a result of these defenses, patients experience repercussions and symptoms.^8^ Concerns about sexual dysfunction, such as reduced or lack of desire or premature ejaculation, may be obscured by fear and worry, as well as emotions of shame or embarrassment, inadequacy, and guilt. These hidden concerns might manifest as a number of symptoms in a range of circumstances, such as physical discomfort, despondency, or marital discontent. As a result, it leads to heated fights inside the family as well as divorce.^9^

Sutherland^10^ indicates that women's sexual troubles have an impact not just on their quality of life but also on their self‐perceptions and interpersonal relationships. According to scholarly studies, sexual irregularities in women are harmful on a physiological, emotional, and social level.^11^, ^12^, ^13^ A man who believes that his wife does not understand him is more likely to engage in self‐defense and self‐care activities. Defenses, in general, are mental procedures that exclude unpleasant emotions—ideas, sensations, or both—from conscious to consciousness.^14^ Defenses might take the shape of ideas, feelings, or both. To make matters worse, our culture is among the few that do not participate in the practice of addressing such topics and difficulties. According to the random effect model (inverse variance technique), the overall prevalence of female sexual dysfunction in Iran was determined to be 43.9%, with a 95% confidence range spanning from 35.0 to 52.8. Desire disorders were found in 42.7% (32.0–53.4), arousal disorders in 38.5% (27.6–49.5), lubrication disorders in 30.6% (22.0–39.2), orgasmic disorders in 29.2% (24.1–34.3), satisfaction disorders in 21.6% (11.5–31.8), and pain disorders in 40.1% (31.8–48.3) of people.^15^ Depressive disorders and their treatment are a major concern due to their high prevalence and high burden in terms of disability, treatment costs, effects on families and carers, and loss of workplace productivity Effectiveness of Short‐Term Dynamic Group Psychotherapy in Primary Care for Patients with Depressive Symptoms. Depression is a leading cause of disability worldwide and contributes significantly to the global disease burden.^16^, ^17^, ^18^, ^19^, ^20^, ^21^, ^22^

When life is seen to be a tough process, psychotherapy treats emotional or psychological suffering as its major emphasis. It reveals how the mind is involved in the effort to face and endure pain, as well as how barriers or systems are built to prevent suffering from occurring. The bulk of these activities occur outside of conscious awareness and involve patterns of observation, thought, emotion, and action. However, because of our limited awareness, most of the time our subconscious attempts to reduce emotional suffering fail, despite being performed numerous times.^23^, ^24^

Psychiatric therapy both supports the person in their distress and works to change how they feel while undergoing treatment. It increases both the therapist's and the patient's understanding of these themes to increase the patient's ability to withstand emotional pain, deal with discontent, and become involved in their experiences.^15^

Habib Davanloo, an Iranian psychotherapist, developed short‐term, intense psychoanalysis at McGill University. It is based on the Freudian psychoanalytic paradigm and employs a more focused kind of analysis. Raven's long‐term analytic technique, which was inefficient, unstructured, and nonspecific, is replaced with a more practical, systematic, and transparent short‐term strategy. “Freud discovered the subconscious, and Davanloo taught himself how to use it for treatment,” Malan explains. “The subconscious was discovered by Freud.”^25^ A variety of studies have been conducted in this field. Parisuz et al.,^26^ for example, investigated the effects of vigorous short‐term dynamic psychotherapy on marital conflict resolution and interpersonal processing in married women who were emotionally prepared to divorce. They discovered that the experimental group had greater interpersonal processing (*F* = 85.43, *p* = 0.01) and lower conflict levels (*F* = 43.93, *p* = 0.01) than the control group. As a result, intensive short‐term dynamic psychotherapy (ISTDP) reduces the incidence of negative sentiments and the resulting marital conflicts, while also improving married couples' interpersonal processing skills.

A total of 115 individuals were treated for depressive symptoms and underwent 75 min of psychotherapy over a 9‐month period (37–39 sessions) (*n* = 70) or standard treatment (*n* = 45). Bros et al.^27^ studied the effectiveness of short‐term group psychotherapy for patients with depressive symptoms in primary care. According to the findings of their study, the mean improvements after treatment were bigger in the psychotherapy group, and the difference in mean benefit between the two groups was statistically significant for the psychotherapy group. According to the data, this medication was effective in treating depressive symptoms.

Solbakken and Abbass^28^ evaluated the personality disorder symptoms and changes that occurred after the treatment of refractory anxiety disorders and depression with short‐term dynamic psychotherapy in 95 patients who had previously been unsuccessful with therapy. According to the findings, this therapy regimen was beneficial for individuals with severe and refractory mental problems. Overall, the data revealed that this therapy regimen had favorable results. Heidarinasab et al.^29^ investigated the efficacy of this therapy in reducing symptom intensity and the frequency of defensive behaviors shown by depressed people.

Psychotherapies that are dynamically oriented are effective treatments for a variety of mental diseases, including depression. When compared to control conditions, a recent meta‐analysis of 23 studies, including short‐term psychodynamic psychotherapy for depression, indicated a significant reduction in depressive symptoms.^30^ Patients with moderate‐to‐severe depression in the community mental health system exhibited moderate‐to‐large effect sizes in favor of brief dynamic psychotherapy over traditional treatment in a randomized pilot study.^22^, ^31^, ^32^, ^33^ However, empirical evidence on the effectiveness of psychodynamic psychotherapy in patients with depressive symptoms in primary care settings is limited.^34^ According to the results, intensive dynamic psychotherapy for a shorter amount of time was beneficial in lowering depressive symptoms and inadequate and immature defensive styles and raising defensive styles after treatment. In light of the need for additional research on marital and family issues in a socially and culturally evolving society like Iran's, approaches that have been less well‐tested in this area, as well as their efficacy on sensitive issues like sexual desire, marital satisfaction, and depression, should be considered. This is due to Iran's social and cultural evolution. As a result, the present study was designed to look into the effect of short‐term psychotherapy on sexual desire and marital satisfaction in depressed women.

## 2 METHODS

### 2.1 Participants

Female patients were referred to the Isfahan City Sexual Disorders Clinic by a licensed psychologist who was conversant with the Diagnostic and Statistical Manual of Mental Disorders, Fifth Edition (DSM‐5). They also had a higher‐than‐average score on the Beck melancholy scale, which led to the diagnosis of sexual desire disorder. The intervention (*n* = 30) and control (*n* = 30) groups each had the same number of people in their samples.

The sample size was determined by the comparison formula for a quantitative feature in two groups as well as its parameters, which are referred to as a 95% confidence coefficient and a 90% test power. Because two individuals are recommended as the minimum number of samples for sexual desire and six individuals are recommended as the minimum number of samples for marital satisfaction, the recommended minimum number of samples should be six individuals. However, the sample size was raised by 10% due to the likelihood of people falling while participating in the study. However, to increase the test's power and generate more reliable results, the sample size for both the intervention and control groups was established at 30 (60 in total). Cleophas et al.^35^ Statistics Applied to Clinical Trials, Springer Netherlands.

$$n=\frac{{\left(Z_{1\frac{\alpha }{2}}+Z_{1-\beta }\right)}^{2}\left(\sigma_{1}^{2}+\sigma_{2}^{2}\right)}{{\left(\mu_{1}-\mu_{2}\right)}^{2}},$$

where *σ*_1_^2^ is the variance of baseline values (at time point 1) in two groups, *σ*_2_^2^ is the variance of follow‐up values (at time point 2) in two groups, 1 − *β* the test power, *α* the type 1 error, *μ*_1_ the average group 1, *μ*_2_ the average group 2, and *Z* the value of the normal variable with confidence level is *α* − 1.

### 2.2 Measures

#### 2.2.1 Beck Depression Inventory (BDI)

The questionnaire's 21 questions are classified into three categories: emotional symptoms, cognitive problems, and physical symptoms. Each item is assigned a score ranging from 0 to 3. The highest possible score is 63, while the lowest possible score is 0. The internal consistency coefficient of the BDI ranged from 0.73 to 0.93, with a mean of 0.86, according to meta‐analysis data. Retest reliability and validity coefficients ranged from 0.48 to 0.86.^28^

#### 2.2.2 DSM‐5 criteria for depressive disorder

The DSM‐5 outlines the following criterion to make a diagnosis of depression. The individual must be experiencing five or more symptoms during the same 2‐week period and at least one of the symptoms should be either (1) depressed mood or (2) loss of interest or pleasure: Depressed mood, loss of interest/pleasure, weight loss or gain, insomnia or hypersomnia, psychomotor agitation or retardation, fatigue, feeling worthless or excessive/inappropriate guilt, decreased concentration, and thoughts of death/suicide.^36^

#### 2.2.3 Enrich Marital Satisfaction Questionnaire (short form)

This scale is comprised of 47 closed‐ended questions, which have been further subdivided into 12 subscales. Each grading scale consists of five questions with no required responses (totally agree, somewhat agree, disagree, totally disagree). Ideal distortions, marital satisfaction, personality difficulties, communication, conflict resolution, money management, leisure activities, sex, children, parenting, and family and friends were among the questionnaire's subscales. On this test, the lowest possible score is 0, and the best possible score is 460. When the score is lower, the person reports less overall pleasure; when the score is higher, the individual reports more overall contentment. Soleimanian^37^ discovered that the dependability coefficient of the 47‐question form and its concurrent validity were both 95% in his study. The Enrich questionnaire's correlation coefficient with components of family satisfaction varied from 0.41 to 0.60, while its correlation coefficient with components of life satisfaction ranged from 0.32 to 0.41, indicating construct validity. The fact that the Enrich questionnaire's many subscales can distinguish between happy and unsatisfied couples indicates that the questionnaire has a high degree of criterion validity.^38^

#### 2.2.4 Women's Sexual Function Assessment Questionnaire

Mohammadi et al.'s^39^ research provided data to support the validity and reliability of the Sexual Function Questionnaire (SFQ). The questionnaire had 19 multiple‐choice questions that assessed women's sexual performance in six distinct categories: desire, sexual arousal, wetness, orgasm, pleasure, and sexual discomfort. The following criteria were considered: desire (from 1 to 5), sexual arousal, wetness, orgasm, sexual pain (from 0 to 5), and sexual pleasure (1–5 or 0). A score of 0 indicates that a person has not engaged in any sexual activity in the recent 4 weeks. Cronbach's *⍺* coefficients have been determined in a number of different internal and external studies, demonstrating the reliability of the scales and subscales and confirming the instrument's excellent dependability. Furthermore, the instrument's validity revealed that there is a significant difference between the mean of the overall scale scores and the scores for each of the domains (*p* = 0.001). According to Mohammadi et al.,^39^ the appropriate cut‐off score on the entire scale for diagnosing sexual dysfunction was 28, and 83% of women with abnormalities were accurately identified, whereas 82% of women without abnormalities were not. Furthermore, 82% of women who had no anomalies received the proper diagnosis.

### 2.3 Procedure

The following criteria were required for participation in the study: the participant had to be married and had been diagnosed with a sexual desire issue by a psychologist. Exclusion from the trial was based on the subject having concurrent treatment with other medications or psychological therapy.

The study population in this specific research attempt consisted of women who were diagnosed by a specialist as having sexual dysfunction and were sent to the Isfahan Marital Care Clinic due to a depressive disorder. Following sample selection based on availability, they were arbitrarily separated into two intervention and control groups (the random assignment was performed based on the case file number of the selected patient; in this instance, even numbers were assigned to the intervention group and individual numbers were assigned to the control group).

To measure participants' sexual dysfunction, sexual satisfaction, and depression, the BDI, the Enrich Marital Satisfaction Questionnaire, and a sexual function assessment were employed. A clinical psychologist with a Tarhe No Institute Certificate in Short‐Term Dynamic Psychotherapy worked with each participant twice a week for 20 sessions of 60 min each at the clinic.

### 2.4 Treatment

The conceptual model was built on Blagys and Hilsenroth's^40^ psychodynamic psychotherapy notions, emphasizing the following areas (with the degree of empirical support stated in parenthesis): (1) a focus on affect and the expression of a patient's emotions (strong support); (2) an exploration of patients' attempts to avoid topics or engage in activities that impede therapy progress (strong support); (3) the identification of patterns in patients' actions, thoughts, feelings, experiences, and relationships (strong support); (4) an emphasis on past experiences (strong support); (5) an emphasis on a patient's interpersonal experiences (strong support); (6) an emphasis on a patient's interpersonal experiences (strong support) (moderate support).

In order for participants to gain a greater understanding, therapists seek to clarify unpleasant experiences, improve communication, and grasp unconscious dynamics. In this way, participants may have a better awareness of themselves, improve interpersonal connections, acquire healthier symptoms, and build more flexible attitudes in everyday life^39^ (Table 1).

**Table 1:** Summary of the content of intensive short‐term dynamic psychotherapy sessions.

Session	Session contents
First session	We discussed the rules for conducting therapeutic sessions and the initial interview with the implementation of the scan sequence, called experimental therapy, to evaluate the patient's initial problem.
Second session	Follow‐up was performed if the experimental treatment responded appropriately. From this moment, that is, from the second session onwards, according to the type of (11) defense, appropriate and effective interventions have been implemented for each patient's defenses. Types of common tactical defenses and effective interventions related to each defense are summarized below.
1. Working with tactical defenses of closed words (to speak in the wrapper), inclusive cover words. Effective intervention: Doubt, challenge, and defense challenge.	
2. Examine specialized words used by patients, tactical defenses of indirect speech, and possible and contagious thoughts. Effective intervention: Challenging patient defenses and speech defining, and challenging defensive and defensive skepticism.	
3. Investigation of intellectual rumination defense and reasoning. Effective interventions: Defining, asking for a definitive response, casting doubt on defense, challenging defense, challenging defense, and blocking defense.	
4. Defenses of rationalization and generalization. Effective Interventions: Defining, damaging, challenging and specifying, and challenging with defense.	
5. Diversion and forgetting tactics. Effective interventions: Block defense and doubt the defense, and challenge the defense.	
6. Deny. Effective interventions: Defining, doubt in defense, and challenging defense.	
7. Exteriorization and ambiguity. Effective interventions: Defining and challenging with defense.	
8. Dodge, obsessive skepticism. Effective interventions: Defining and challenging with defense.	
9. Bodybuilding and acting as a defense against emotions. Effective intervention: Enlightenment.	
10. Rebellion, disobedience, defensive crying, and the specter of backward defenses. Practical interventions: Confrontation, challenge, direct involvement, and enlightenment.	
11. Talking instead of touching emotions. Nonverbal symptoms. Obedience‐passivity. Effective Interventions: Defining. Doubt in defense, and challenge with defense. Challenge.	
At the end of the last session, the follow‐up program, especially the posttest time, was announced, the patients were thanked, and the end of the sessions was announced.	

**Figure 1:**
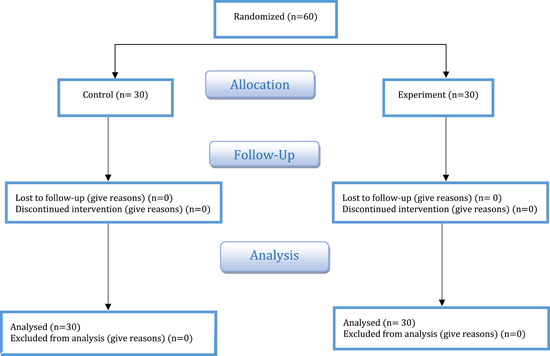
CONSORT flow diagram of participant enrollment.

The sample size was determined by the comparison formula for a quantitative feature in two groups as well as its parameters, which are referred to as a 95% confidence coefficient and a 90% test power. Because two individuals are recommended as the minimum number of samples for sexual desire and six individuals are recommended as the minimum number of samples for marital satisfaction, the recommended minimum number of samples should be six individuals. However, the sample size was raised by 10% due to the likelihood of people falling while participating in the study. However, to increase the test's power and generate more reliable results, the sample size for both the intervention and control groups was established at 30 (60 in total) (Figure 1).

### 2.5 Statistical analysis

To determine the normal distribution of data, the Kolmogorov–Smirnov test was applied. In addition to descriptive statistics, the univariate analysis of covariance (ANOVA) was used to assess the data that was gathered in light of the study objectives. All tests were analyzed through the SPSS statistics software V.24 at the significance level of *p* < 0.001.

### 2.6 Ethics approval

Kermanshah University of Medical Sciences conducted this investigation (research number 96338). And the Kermanshah University of Medical Sciences Ethical Committee authorized this research (KUMS.REC.1396.259). The study is included in the Iranian Registry of Clinical Trials (IRCT2017082425433N4).

## 3 RESULTS

There were around 55% of individuals who were between the ages of 18 and 28, 30% between the ages of 29 and 39, and 15% between the ages of 40 and older. In terms of education, 42.54% only had a high school diploma or a lesser level of education, 28.64% had technical education after high school, and 28.82% had a college degree.

Both the experimental and control groups reported the mean and standard deviation of participants' ratings of marital satisfaction, sexual function, and depression. As seen in Table 2, marital satisfaction, sexual function, and depression mean in the experimental group (short‐term dynamic psychotherapy) at posttest stage were significant changes (*p* < 0.001). But no significant change was observed in the control group in pre‐ and posttest stages. In the follow‐up phase, the results were stable.

**Table 2:** Mean and standard deviation of variables in pretest and posttest.

Variable	Group	Pretest		Posttest		Follow‐up		
		Mean	SD	Mean	SD	Mean	SD	*p* Value
Marital satisfaction	Short‐term dynamic psychotherapy	140	10.45	127.50	6.22	125	5.72	0.001
	Control	135.30	6.60	139.11	10.41	138.51	10.43	0.137
Sexual desire	Short‐term dynamic psychotherapy	31.90	21.27	59	5.67	52.68	4.87	0.002
	Control	33.70	9.20	33.40	19.12	32.86	19.43	0.948
Depression	Short‐term dynamic psychotherapy	23.90	5.85	4.10	2.99	4.10	2.99	0.001
	Control	24.20	2.930	24.80	5.49	23.30	4.89	0.874

Besides, before conducting a multivariate analysis of variance (MANOVA), Levin's test was used to observe the defaults of this analysis. Note that if two groups are equal or greater than 40 subjects, it is not necessary to observe the icnadiova of the equality of equivalence of variance (Levine), and consistency of covariance (Box's *M* test). Here, the homogeneity of variance was observed. Therefore, the use of multivariate variance analysis was possible. The results of Leven's test are shown in Table 3 that shown data normally and the defaults have been met.

**Table 3:** Results of Leven's test regarding the equality of variances in marital satisfaction, sexual desire, and depression scores.

Variables	*F*	*df*_1_	*df*_2_	Significant
Marital satisfaction	3.40	1	58	0.08
Sexual desire	4.44	1	58	0.04
Depression	8.19	1	58	0.01

The results of MANOVA revealed that there was a significant difference between the short‐term dynamic psychotherapy and control groups in terms of marital satisfaction, sexual function, and depression (*p* < 0.001), and the statistical power of 86% also showed that the sample size was sufficient for analyses (Table 4).

**Table 4:** Results of multivariate analysis of variance for comparing the mean posttest scores of variables in the study groups.

*n*	Value	*F*	Hypothesis *df*	Error *df*	Significant	*η* ^2^
Pillai's trace	0.86	19.47	3	29	0.001	0.86
Wilks's lambda	0.13	19.47	3	29	0.001	0.86
Hoteling's trace	6.49	19.47	3	29	0.001	0.86
Roy's largest rot	6.49	19.47	3	29	0.001	0.86

ANCOVA was conducted to find out the difference observed. Considering the calculated effect size, 51% of the total variances of experimental and control groups was the result of the effectiveness of the independent variable. Moreover, the statistical power of the test was 0.80, which means that the test was able to reject the null hypothesis with a power of 51% (Table 5).

**Table 5:** Results obtained from univariate analysis of covariance (ANOVA).

Dependent variable	Mean square	Degrees of freedom	*F*	Significant	*η* ^2^
Marital satisfaction	511.91	1	11.72	0.006	0.51
Sexual desire	1800.39	1	13.64	0.004	0.55
Depression	1457.93	1	62.52	0.001	0.85

Abbreviation: ANOVA, analysis of variance.

## 4 DISCUSSION

The goal of this study was to determine how a short, intensive kind of psychotherapy affected the amount of marital satisfaction and sexual desire experienced by depressed women. After adjusting for pretest scores, the analysis of variance on pretreatment and treatment scores revealed a significant difference between the experimental and control groups in the variables of women's marital satisfaction, sexual performance, and depression (*p* = 0.05). This difference was detected in marital satisfaction, sexual performance, and depression. In other words, rigorous short‐term dynamic psychotherapy has been proven to boost sexual performance and marital satisfaction in women, as well as lessen depressive symptoms in women who suffer from depression, based on average posttest scores for sexual performance and marital satisfaction.

This work adds weight to previous research^29^, ^41^, ^42^ by demonstrating that dynamic psychotherapy may reduce depressive symptoms in a very short period of time. Another study discovered that psychiatric therapy has the potential to increase marital satisfaction in depressed women.^43^, ^44^ The clinical examination, on the other hand, demonstrated that patients were able to discuss their differences with their peers. It improved their relationships with others by effectively reducing arguments; they were able to calmly convey their views and feelings; and they were successful in reaching their objectives in a less stressful communication environment. They reported an increase in marital happiness as a direct consequence of this.

In an effort to explain these results, one option is that psychodynamic psychotherapies are based on the assumption that the source of mental illnesses is emotional conflict. This method confronts the patient with emotional issues that have formed in the past, present, and as a consequence of their interaction with the therapist, using techniques like guided imagery and progressive desensitization. The patient is able to settle her emotional conflicts by recognizing and experiencing feelings that are both stimulating and inhibiting.^41^ Intensive short‐term dynamic psychotherapy, on the other hand, has been shown to improve patients' marital satisfaction and sexual performance while also reducing irrational beliefs, cognitive avoidance, depressive symptoms, negative mood, social anxiety, uncompromising defensive styles, and neuroticism.^26^, ^45^

Based on the “conflicting relationship premise,” Ahmadi et al.^46^ evaluated the effect of short‐term dynamic psychotherapy on couples' marital satisfaction. According to their findings, short‐term dynamic psychotherapy increased marital satisfaction while decreasing the frequency of marital issues. Jarareh and Talepasand^47^ investigated the impact of tactical defense management on the short‐term dynamic psychotherapy process and its impact on marital happiness in couples. They discovered that using therapeutic treatments in tactical defense might significantly boost marital happiness. Mobassem et al.^48^ discovered that short‐term dynamic psychotherapy was successful in reducing the amount of conflict in couples. Kramer et al.^49^ explored the impact of the emotional processing of sadness on adjustment disorder using a dynamic psychotherapy paradigm. According to their findings, therapy was successful in lowering emotions of despair and increasing levels of patience. Furthermore, Johansson et al.^50^ revealed that short‐term psychodynamic therapy increases patients' ability to tolerate emotional pain and manage dissatisfaction, as well as their capacity to reflect on and be interested in their experiences in numerous aspects of life. This is because patients are more able to reflect on and inquire about their experiences. The findings of this study correspond with the findings of earlier investigations, indicating that this study is credible.

The clinical evaluation reveals that boosting interpersonal interaction and improving clients' abilities in the treatment process will increase the amount of time spent discussing the husband and wife's marital relationship. As a result, a constructive dialog is created about their daily duties or activities, as well as their interests, feelings, wants, wishes, and expectations, and in general, good and consistent communication enhances their level of pleasure. Referees may successfully manage their issues and offer their viewpoints on a number of themes, such as financial management, parenting, and communication, provided they can explain their needs in a manner that does not require them to anticipate others repeating their previous comments. They are unconcerned with the possible consequences of discussing their sexual orientation and religious views freely and honestly in the face of their family and friends. Furthermore, they can tolerate and bear the other person's criticisms and objections with less grief and misery. Evolved communication patterns, as well as emotion management and control, which are frequently obtained as a result of resolving intrapersonal and interpersonal conflicts, as well as the broad insight gained during the treatment process about the fields of behavior and emotions related to them, improve individual performance and satisfaction levels.

The findings also revealed that short‐term dynamic psychotherapy sessions increased sexual desire in women with depression as compared to the control group. When compared to the control group, this was the case. Short‐term dynamic psychotherapy results from Fooladi et al.,^51^ Mohamadzadeh and Hoseini,^52^ Mami and Heidary,^42^ and Driessen et al.^30^ can be summarized as a reduction in irrational beliefs and cognitive avoidance, as well as a decrease in depressive symptoms and negative moods, social anxiety, and disorganized and neurotic defensive behavior. Furthermore, they increase patients' emotional expressiveness as well as their desire for sexual relations. When these findings are interpreted in a certain way, it is argued that a number of negative attitudes that are antithetical to indices and components of quality of life reduce the extent to which people suffering from depression experience sexual pleasure. Women suffering from depression not only have decreased sexual desire, but they also do not participate in sexual activity with their husbands due to psychological and emotional difficulties. Having a personal connection with one another and the ability to convey sentiments may help minimize the negative impressions held by this group of women while increasing their readiness to participate in sexual activities.

Individuals may articulate their thoughts and better explain their needs to their spouses owing to training offered in rigorous, short‐term, dynamic psychotherapy sessions that reveal, block, challenge, and recognize self‐defense resistance. People are given the opportunity to better express their sentiments and get a better awareness of their own difficulties, resulting in the emergence of this reality. They may respond positively to their desires and needs if they first recognize their weaknesses and then fight those deficiencies.^51^ Patients who attended therapy sessions were aware that their negative emotions contributed to the development of the disease and its recurrence. The underlying process studied in psychopathology emphasizes the idea that unbearably unpleasant sensations are repressed and not brought into conscious awareness. These ideas and sentiments, on the other hand, are not unpleasant and may be met with curiosity and understanding. They are capable of creating soothing and healing effects when brought to awareness and properly understood.

The intense, rapid type of dynamic psychotherapy emphasizes maintaining a positive attitude in life. It also improves a patient's ability to cope with stressful situations, making them more adaptable in the face of problems and marital disputes, and ensuring that they do not lose sexual desire. The ill person makes an attempt to keep her emotions under control and maintains a positive attitude about her present situation. She also pushes herself to believe that she is better capable of adapting to the hardships of married life, which leads to a greater sense of sexual self‐esteem. It is possible to say that a significant portion of the marital relationship is built on emotional and emotional issues, as well as the inability to express these feelings in a timely and appropriate manner and the inability to remember previous pleasurable sexual experiences with the spouse, which is common in 80% of these. It is feasible to state that emotions and emotional concerns form a big part of the marriage connection. It eventually leads to emotional divorce and sexual dysfunction in this group of patients, which leads to an emotional retreat, which leads to a loss of intimacy. As a result, rigorous short‐term dynamic psychotherapy heals the patients' internal concerns while also locating and resolving the causes of their sexual performance issues. Because these difficulties may be connected to the patients' emotions and internal concerns, rigorous short‐term dynamic psychotherapy addresses them as well.

The intervention procedure, on the other hand, has the potential to offer patients a feeling of emotional relaxation and psychological comfort, both of which have a favorable influence on the patient's capacity to boost their sexual function. The initial indicator of depression is a decrease in libido, which is followed by weight‐change symptoms such as anorexia and sleepiness, as well as a disturbance in the menstrual cycle. The modern strategy for treating severe depression is based on integration (psychotherapy and pharmacotherapy). The purpose of sexual therapy is to modify some characteristics of a person's sexual behavior. Individuals must be free of negative sentiments and excessive cognitive control to have a practical sexual function (which enhances cohesion and relationship happiness).^53^

After participating in rigorous short‐term dynamic psychotherapy based on the Divanloo theory, this study highlighted and challenged the defenses of women suffering from depression. Brain stimulation was used to detect intellectual risks, illogical conceptions, thoughts, and worries, as well as to avoid sexual harassment, stress, rejection, dread, worry, and despair. They now realized the emotional pain and bodily wounds they had suffered in the past. They reported feeling better after emotionally evacuating themselves.

As a result, short‐term, dynamic active psychotherapy focusing on sexual function is an effective and trustworthy therapeutic paradigm, and it may improve the marital satisfaction of depressed couples. On the other hand, it has the significant benefit of not requiring the couple to visit for therapy; nonetheless, when one of the couples attends treatment, significant improvements occur, marital satisfaction increases, and performance in the sub‐scales improves.

### 4.1 Limitations

This study, like other types of research, has certain flaws. Because the research sample was comprised of depressed women, it is crucial to take care when attempting to apply the findings of this study to women suffering from other conditions. Furthermore, the sample population of this study exclusively comprised female participants from the city of Isfahan. Furthermore, the researchers did not track the therapeutic results throughout the experiment. It has been proposed that future statistical population selection studies look at women suffering from depression in a range of settings. This is due to the cultural and social contrasts between these two locations.

The use of an available sample approach, the limiting of participants to females, a lack of appropriate time and crucial teamwork to follow up and analyze the durability of treatment effects, and the research being done entirely on women were all flaws in this study. In general, counsellors and psychotherapists consider that intense short‐term dynamic psychotherapy is an appropriate intervention in the counselling center for improving sexual performance and marital satisfaction in women suffering from depression. This is because it is regarded as an effective remedy for improving sexual performance and marital satisfaction in depressed women.

Furthermore, the researchers did not track the therapeutic results throughout the experiment. In light of this, it is highly advised that larger samples be employed in future research to investigate some of the potential variables that may have an impact on the control group. Furthermore, a variety of additional ways of gathering information centered in the community, such as interviews, might be utilized to test this group to widen the range of the information acquired. It is also suggested that research into additional intervention approaches and features of this business be done and that the findings be compared and assessed together. In general, counsellors and psychotherapists should prescribe rigorous short‐term dynamic psychotherapy as a suitable strategy to improve sexual function, marital satisfaction, and depression in women suffering from depression.

## 5 CONCLUSION

The findings of this study provide preliminary evidence that ISTDP is more successful than minimal contact in treating depression in people. It adds to the studies that have previously proven the treatment to be clinically helpful as well as cost‐effective, with benefits that last for a long time. The limitations of this study must be addressed in future ISTDP research, including this demography as well as the introduction of formal cost–benefit analyses. Furthermore, it must explore the specific impacts of patient variables, therapist factors, and therapeutic factors (e.g., emotional experiences). A study of this kind might possibly contribute to ongoing research initiatives.

## AUTHOR CONTRIBUTIONS

**Arash Ziapour**: Conceptualization; methodology; supervision; writing—original draft; writing—review and editing. **Arezou Hajiazizi**: Data curation; validation. **Maryam Ahmadi**: Data curation; formal analysis; visualization. **Fateme Dehghan**: Conceptualization; methodology; writing—original draft; writing—review and editing.

## CONFLICT OF INTEREST STATEMENT

The authors declare no conflict of interest.

## TRANSPARENCY STATEMENT

The lead author Arash Ziapour affirms that this manuscript is an honest, accurate, and transparent account of the study being reported; that no important aspects of the study have been omitted; and that any discrepancies from the study as planned (and, if relevant, registered) have been explained.

## ETHICS STATEMENT

The study was approved by the Research Ethics Committee of Kermanshah University of Medical Sciences (IR.KUMS.REC.1399.1105). Written informed consent was obtained from all group members. Consent to submit has been received explicitly from all coauthors, as well as from the responsible authorities—tacitly or explicitly —at the institute/organization where the work has been carried out before the work is submitted. The purpose of this research was completely explained to the participants through the cover page of the questionnaire, and they were assured that their information would be kept confidential by the researcher. Informed consent from the participants was acquired as they agreed to participate in the study by reviewing the questionnaire's cover page and clicking on the provided link. Furthermore, for participants younger than 18 years of age, the participant was asked for the consent of the parent or guardian.

## Data Availability

The data supporting the findings in this study will be made available through the corresponding author upon reasonable request. The corresponding author had full access to all of the data in this study and takes complete responsibility for the integrity of the data and the accuracy of the data analysis.
